# Evolutionary analysis of the *SUB1* locus across the *Oryza* genomes

**DOI:** 10.1186/s12284-016-0140-3

**Published:** 2017-02-07

**Authors:** Railson Schreinert dos Santos, Daniel da Rosa Farias, Camila Pegoraro, Cesar Valmor Rombaldi, Takeshi Fukao, Rod A. Wing, Antonio Costa de Oliveira

**Affiliations:** 1 Technology Development Center (CDTec), Universidade Federal de Pelotas, Pelotas, Brazil; 2 Plant Genomics and Breeding Center (CGF), Universidade Federal de Pelotas, Pelotas, Brazil; 3 Crop and Soil Environmental Sciences, Virginia Tech, Blacksburg, USA; 4 The School of Plant Sciences, Ecology & Evolutionary Biology, Arizona Genomics Institute, Tucson, USA

**Keywords:** IOMAP, *Oryza*, *Leersia perrieri*, Submergence tolerance, *SUB1* genes

## Abstract

**Background:**

Tolerance to complete submergence is recognized in a limited number of Asian rice (*Oryza sativa* L.) varieties, most of which contain submergence-inducible *SUB1A* on the polygenic *SUBMERGENCE-1* (*SUB1*) locus. It has been shown that the *SUB1* locus encodes two *Ethylene-Responsive Factor* (*ERF*) genes, *SUB1B* and *SUB1C*, in all *O. sativa* varieties. These genes were also found in *O rufipogon* and *O nivara*, wild relatives of *O. sativa*. However, detailed analysis of the polygenic locus in other *Oryza* species has not yet been made.

**Findings:**

Chromosomal location, phylogenetic, and gene structure analyses have revealed that the *SUB1* locus is conserved in the long arm of chromosome 9 in most *Oryza* species. We also show that the *SUB1A-like* gene of *O. nivara* is on chromosome 1 and that *Leersia perrieri*, a grass-tolerant to deep-flooding, presents three *ERF* genes in the *SUB1* locus.

**Conclusion:**

We provide here a deeper insight into the evolutionary origin and variation of the *SUB1* locus and raise the possibility that an association of these genes with flooding tolerance in *L. perrieri* may exist.

**Electronic supplementary material:**

The online version of this article (doi:10.1186/s12284-016-0140-3) contains supplementary material, which is available to authorized users.

## Findings

Abiotic stresses such as flooding can prevent plants from attaining their full genetic potential for growth and reproduction. In Asia, the leading producers of rice (*Oryza sativa* L.), many flood-prone lands are used for rice production, reason why so many studies are developed aiming to understand and overcome flooding response mechanisms.

A major quantitative trait locus (QTL) called *SUBMERGENCE-1* (*SUB1*) is responsible for conferring submergence tolerance to the rice landrace Flood Resistant 13A (FR13A). Detailed sequence analysis revealed that the locus encodes a variable cluster of up to three *Ethylene-Responsive Factors* (*ERF*): *SUB1A*, *SUB1B* and *SUB1C* (Fukao et al., 2006; Xu et al., 2006).

All surveyed *O. sativa* varieties contain *SUB1B* and *SUB1C*, whereas *SUB1A* is present only in a limited number of accessions. Genotypes containing submergence-inducible *SUB1A* restricts underwater ethylene production, which maintains mRNA and protein accumulation of gibberellic acid (GA) signaling repressors during submergence (Fukao and Bailey-Serres, 2008; Fukao et al., 2006; Singh et al., 2014). *SUB1A*-mediated inhibition of GA responsiveness consequently represses genes required for starch and sucrose catabolism. This strategy characterized by reduced plant elongation in order to preserve the sugars needed for regrowth when water recedes is called ‘quiescence strategy’ and it is being very useful in plant breeding (Neeraja et al., 2007; Fukao and Bailey-Serres, 2008; Fukao et al., 2006; Septiningsih et al., 2009; Septiningsih et al., 2013; Iftekharuddaula et al., 2015).

Wild rice species generally lack agriculturally important traits. However, they carry many desirable genes that have been lost in cultivated rice. Here, we report that previous analyses made by Fukao et al. (2009) and Niroula et al. (2012) are now extended across large part of *Oryza* phylogeny using sequencing data from the International *Oryza* Map Alignment Project (IOMAP) consortium (Stein et al: Sequence of 11 rice-related species unveils the Oryza pan-genome and the origin of genetic innovation, submitted; Jacquemin et al., 2013): *O. sativa* L. ssp *indica* (AA), *O. sativa* L. ssp *japonica* (AA), *O. rufipogon* Griff. (AA), *O. nivara* Sharma et Shastry (AA), *O. glumaepatula* Steud. (AA), *O. glaberrima* S. (AA), *O. barthii* A. Chev. (AA), *O. meridionalis* Ng. (AA), *O. punctata* Kotschy ex Steud. (BB), and *O. brachyantha* Chev. et Roehr (FF).

Our genomic analysis identified 58 *SUB1*-like genes (Additional file 1: Table S1). The alignment of the AP2 domains with the signature amino acids alanine and aspartic acid at positions 13 and 18, respectively, of the AP2 domain, a characteristic of the B2 subgroup of ERF proteins, can be seen in Additional file 2: Figure S1.

**Fig. 1:**
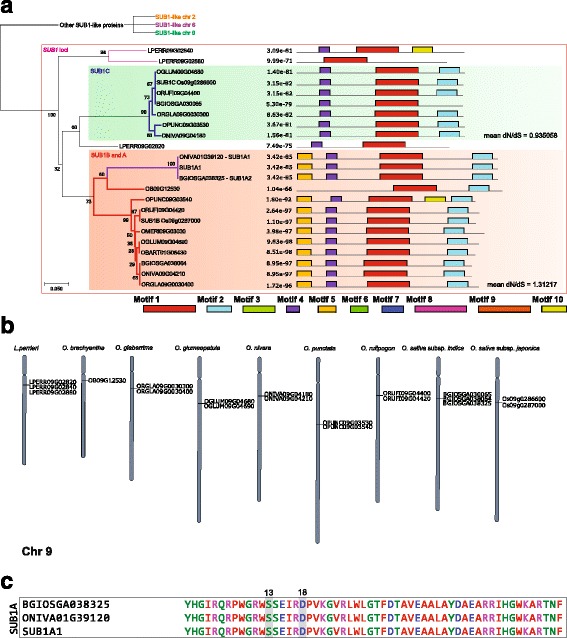
*SUB1* analyses across the *Oryza* genomes. **a** Phylogenetic tree generated from the protein alignment of *SUB1-*like genes identified of all species analyzed, genes in red are *SUB1B-*like genes and green are *SUB1C-*like. On the right side of the tree, amino acid motifs are represented. Full version and motif logos are available in Additional file 3: Figure S2 and Additional file 11: Figure S8. **b** Chromosomal location of the *SUB1*-like loci in each *Oryza* genome (chromosomes 1 and 9). Full version available in Additional file 4: Figure S3. **c** Alignment of the AP2 domains of *SUB1A-*like genes in rice relatives

Of nine species, six of them such as *O. rufipogon* (AA), *O. nivara* (AA), *O. glumaepatula* (AA), *O. glaberrima* (AA), *O. sativa* (AA), and *O. punctata* (BB) contained both *SUB1B-* and *SUB1C*-like genes side by side on chromosome 9 (Chr 9), as shown in Fig. 1a and b, which is consistent with the observations in multiple accessions of *O. sativa* (AA) (Singh et al., 2010; Xu et al., 2006). According to its position in the genome and to the proximity between these genes we first divided these *SUB1*-like genes into two large supergroups: “Other SUB1-like proteins” and “*SUB1* loci”, as seen in Fig. 1a.

We further divided the supergroup called “Other SUB1-like proteins”, which is formed by longer proteins, into three groups: "*SUB1*-like Chr 2" (*SUB1*chr2), "*SUB1*-like Chr 6" (*SUB1*chr6) and "*SUB1*-like Chr 9" (*SUB1*chr9) (Additional file 3: Figure S2). *SUB1*chr2 consists of genes on Chr 2, while *SUB1*chr6 group consists of genes at the Chr 6 with a deletion of motif 10, and an inverted region, with motif 7 after motif 2. The third group, SUB1chr9, is present on Chr 9 and genes of this group possess the motif 9 preceding motif 3. *O. glumaepatula* showed the most distinct arrangement of motifs with a deletion covering motifs 5 and 4 which made the gene much shorter in this species. *O. brachyantha* presented a gene on SUB1chr9 that resembles those grouped as SUB1chr2.

The *OsERF#70* (*Os02g0782700*), which is the *SUB1*chr2 gene found in *O. sativa* is already known to interact with a Myc transcription factor called OsBP-5, coregulating the expression of the *waxy* gene (Zhu et al., 2003). Such information shows that the genes contained in the supergroup "Other *SUB1*-like genes" have the potential to interact with Myc factors. The similarities of these longer genes with those found in the *SUB1* locus should be better studied in order to analyze their significance in altering plant tolerance to stresses and/or other important traits that may bring interesting insights about ERF evolution or that may be of interest of breeders.

The second large supergroup, which we called “*SUB1* loci” (Fig. 1a), is formed by shorter genes, on the long arm of Chr 9, near the centromere (Fig. 1b). Most of these genes are positioned in a similar way as the *SUB1* locus described by Xu et al. (2006). Figure 1b and Additional file 4: Figure S3 show the positions of these genes on chromosomes of different genomes.

Interestingly, we also found *SUB1A-* and *SUB1B*-like genes on Chr 1 of *O. nivara* (*ONIVA01G39120*) and *O. barthii* (*OBART01G06430*), respectively. To identify possible translocation events related to these specific loci we aligned regions corresponding to 50 Kb upstream and 50 Kb downstream of *ONIVA01G39120* and *OBART01G06430* to SUB1 loci (Chr 9) of each *Oryza* species. The alignments show no similar regions when SUB1 loci are compared to *ONIVA01G39120*, nevertheless we can identify an orthologous region (~8.5 Kb) when SUB1 loci of all genome AA species are compared to *OBART01G06430* (Additional file 5: Figure S4). The region was searched for the presence of transposable elements (TEs) that were possibly involved with this event, but no clear mechanism involving TEs was detected.

It is also interesting to highlight that the genomes of *O. meridionalis* (AA), *O. barthii* (AA) and *O. brachyantha* (FF) encoded only one *SUB1-*like gene, all of them more related to *SUB1B* than to *SUB1C*.

Three genes closely related to *SUB1* locus were found in Chr 9 of *Leersia perrieri* (A. Camus) Launert. (*LPERR09G02820*, *LPERR09G02840* and *LPERR09G02880*) demonstrating the presence of duplication events in the onset of *SUB1B* and *SUB1C*, which occurred after the divergence of *Oryzeae* from other grasses. This duplication event seems to have occurred before the speciation of *L. perrieri*.

*SUB1A*-like genes were not detected in the genomes of these rice relatives, except for *O. nivara* (Fig. 1c). An alignment of the gene found by Niroula et al. (2012) (cultivar IRGC:80507), *ONIVA01G39120* (cultivar IRGC:100897) and original *SUB1A-1* demonstrates that, despite differences in the C-terminal region, the two *O. nivara* sequences are probably variants (alleles) from the same locus. *O. brachyantha* also presented a gene that was similar to *SUB1* (*OB09G12530*), but the absence of a serine at position 13, and other differences along their sequence prevented its classification as a *SUB1A*-like gene. It is interesting to notice that the *SUB1A*-like genes found in *O. nivara* (*ONIVA01G39120*) and *O. barthii* (*OBART01G06430*), were not on Chr 9, but on Chr 1.

Constitutive and conditional expression of *SUB1A* significantly increases tolerance to complete submergence in *O. sativa* (Fukao et al., 2006; Xu et al., 2006). Detailed genetic survey demonstrated that submergence-tolerant accessions of *O. sativa*, *O. nivara* and *O. rufipogon* possess *SUB1A-1* which is highly induced by submergence, suggesting that *SUB1A* arose *via* tandem duplication prior to rice domestication (Niroula et al., 2012; Singh et al., 2010). In this study, we surveyed the entire genome sequences of nine rice relatives to identify orthologs of *SUB1A* as well as *SUB1B* and *SUB1C*.

Chromosomal location of the Chr 9 *SUB1* locus was highly conserved in *O. nivara*, *O. rufipogon*, *O. glumaepatula* and *O. glaberrima* which had a locus deviation smaller than 2.0 Mb. The deviation of *O. punctata*, which also had a similar conserved structure in the long arm of Chr 9, was a little larger reaching 3.06 Mb (Fig. 1b).

To estimate how natural selection is acting on the coding sequences of *SUB1C*- and *SUB1B*-like genes, the ratio of synonymous/non-synonymous mutations (dN/dS) was calculated (Fig. 1a and Additional file 6: Figure S5). The mean dN/dS is 1.312 (positive selection) for *SUB1B-* and 0.935 (negative selection) for *SUB1C*-like genes. The freedom that *SUB1B*-like genes have to be modified may have been essential to the emergence of *SUB1A*-like genes, which are extremely close to *SUB1B* genes.

The analysis of the upstream sequences (-1,500 bps) of *SUB1A*-like genes shows that the gene found in *O. nivara* must have a similar regulation to the *SUB1A-1* allele responsible for tolerance to submergence in *O. sativa* (Additional file 7: Figure S6). The transcription factor binding sites (TFBSs) found in the upstream region of *ONIVA01G39120* (Additional file 8: Table S2), together with the location of polymorphisms found when comparing *SUB1A-1* and *SUB1A-2* alleles from *O. sativa* (Additional file 9: Figure S6) shows that diverse transcription factors, among them WRKY and ERF, may be important regulators of this gene in submergence conditions. This conclusion is based on the fact that binding sites for these TFs were detected in families where differences between the promoter regions of *SUB1A-1* (upregulated by submergence) and *SUB1A-2* (non-upregulated by submergence) alleles were found. Now we should further investigate the possibly tolerant phenotype resulting from the introgression of *ONIVA01G39120* into *O. sativa* elite cultivars and how is it regulated since this constitutes a very relevant information to breeders.

Integration of genome sequences with RNAseq data allowed us to construct gene structure models of *SUB1*-like genes isolated from these rice relatives (Additional file 10: Figure S7). Gene structure analysis revealed the presence of an intron inside the coding sequence (CDS) of *SUB1B*-like genes, except for *O. glaberrima*, *O. rufipogon* and *O. glumaepatula*.

*SUB1C* alleles of *O. sativa* contain an intron in the 3’ untranslated region (UTR). However, our gene structure analysis based on RNAseq data did not detect 3' UTR sequences of *SUB1C-*like genes (Additional file 10: Figure S7). Therefore, the intron in the 3’ UTR region of *SUB1C-*like genes was sought within 1.5 kb downstream of the stop codon in these genes and regions highly similar to the *O. sativa* intron were found in *ORUFI09G04400*, *ONIVA09G04180*, *OGLUM09G04680*, *OGLAB09G030300* and *OPUNC09G03530* indicating that most *SUB1C*-like genes identified in *Oryza* species possess traces of an intron in their terminal region (Additional file 11: Table S3).

In summary, here we present *SUB1*-like genes identified in wild rice species through genome-wide sequence analysis. Our chromosomal location, phylogenetic, and gene structure analyses have revealed that the *SUB1* locus is conserved in the long arm of Chr 9 in most *Oryza* species with AA-, BB- and FF-genomes. We also showed that the *SUB1A*-like gene of *O. nivara* is on Chr 1 and that *L. perrieri*, a grass-tolerant deep-flooding, presents three *ERF* genes in *SUB1* locus, similar to that found in flooding tolerant rice. This finding raises the possibility that an association of this gene with flooding tolerance in *L. perrieri* exists. Along with genes found in Chr 1, *SUB1*-like genes found in Chr 2 and Chr 6 represent some new interesting information. These genes should now be further investigated in order to check their biological and agricultural significance. Allelic survey of diverse ecotypes in wild rice species is necessary to determine the evolutionary origin and biological significance of *SUB1A* in the genus *Oryza* (Additional file 12: Table S4, Additional file 13: Table S5 and Additional file 14).

## Additional files

Additional file 1: Table S1.
*SUB1*-like genes identified in *Oryza* species. (XLSX 22 kb)
Additional file 2: Figure S1. Alignment of the AP2 domains of *SUB1-*like genes in rice relatives. Signature amino acids specific to B2 subgroup of ERF proteins are highlighted. (PDF 403 kb)
Additional file 3: Figure S2. Phylogenetic tree generated from the protein alignment of *SUB1-*like genes identified of all species analyzed, genes in red are *SUB1B-*like genes and green are *SUB1C-*like. On the right side of the tree, amino acid motifs are represented. Motif logos are available in Additional file 11: Figure S8. (PDF 1078 kb)
Additional file 4: Figure S3. Chromosomal location of the *SUB1*-like loci in each *Oryza* genome (chromosomes 1 and 9). (PDF 457 kb)
Additional file 5: Figure S4. Alignment of *OBART01G06430* genomic region (first line) to SUB1 loci (Chr 9) regions of *Oryza* species of AA genome. Same colors represent collinear blocks between genomes. (PDF 174 kb)
Additional file 6: Figure S5. Phylogenetic tree, motifs and evolutionary pressures on proteins quantified by the ratio of substitution rates at non-synonymous and synonymous sites (dN/dS) for *SUB1B-*like *and SUB1C-*like genes. (PDF 582 kb)
Additional file 7: Figure S6. Alignment of the promoter region (1,500 bps upstream) of *SUB1A*-like genes, showing polymorphisms found when comparing *SUB1A-1* and *SUB1A-2* alleles. (PDF 2215 kb)
Additional file 8: Table S2. Transcription Factor Binding Sites (TFBSs) in the promoter regions (-1,500 bps upstream) of *SUB1A*-like gene of *O. nivara*. Regions polymorphic when comparing (XLSX 212 kb)
Additional file 9: Figure S7. Structure of the *SUB1*-like genes in each of the different species. (PDF 399 kb)
Additional file 10: Table S3. BLAST analysis searching the 3’UTR intron in *SUB1C*-like genes. (XLSX 12 kb)
Additional file 11: Figure S8. Motifs found on the analysis of the amino acid sequences of *SUB1*-like genes. (PDF 416 kb)
Additional file 12: Table S4. Genes with e-value below 1e^−25^ found by BLAST. (XLSX 21 kb)
Additional file 13: Table S5. Gene structure information (General feature format) about each of the analyzed genes.. (XLSX 239 kb)
Additional file 14: Methods. (DOCX 24 kb)

## Abbreviations

CDS: Coding sequence
dN/dS: Ratio of synonymous/non-synonymous mutations
*ERF*: *Ethylene-Responsive Factor*
FR13A: Flood Resistant 13A
GA: Gibberellic acid
IOMAP: International *Oryza* Map Alignment Project
QTL: Quantitative trait locus
*SUB1*: *SUBMERGENCE-1*
UTR: Untranslated region
