# Supplementary figures and images for: Evolutionary analysis of the SUB1 locus across the Oryza genomes
Source: Rice (N Y). 2017 Feb 7;10:4. doi: 10.1186/s12284-016-0140-3 (PMC5296262; doi:10.1186/s12284-016-0140-3)

Other SUB-like proteins

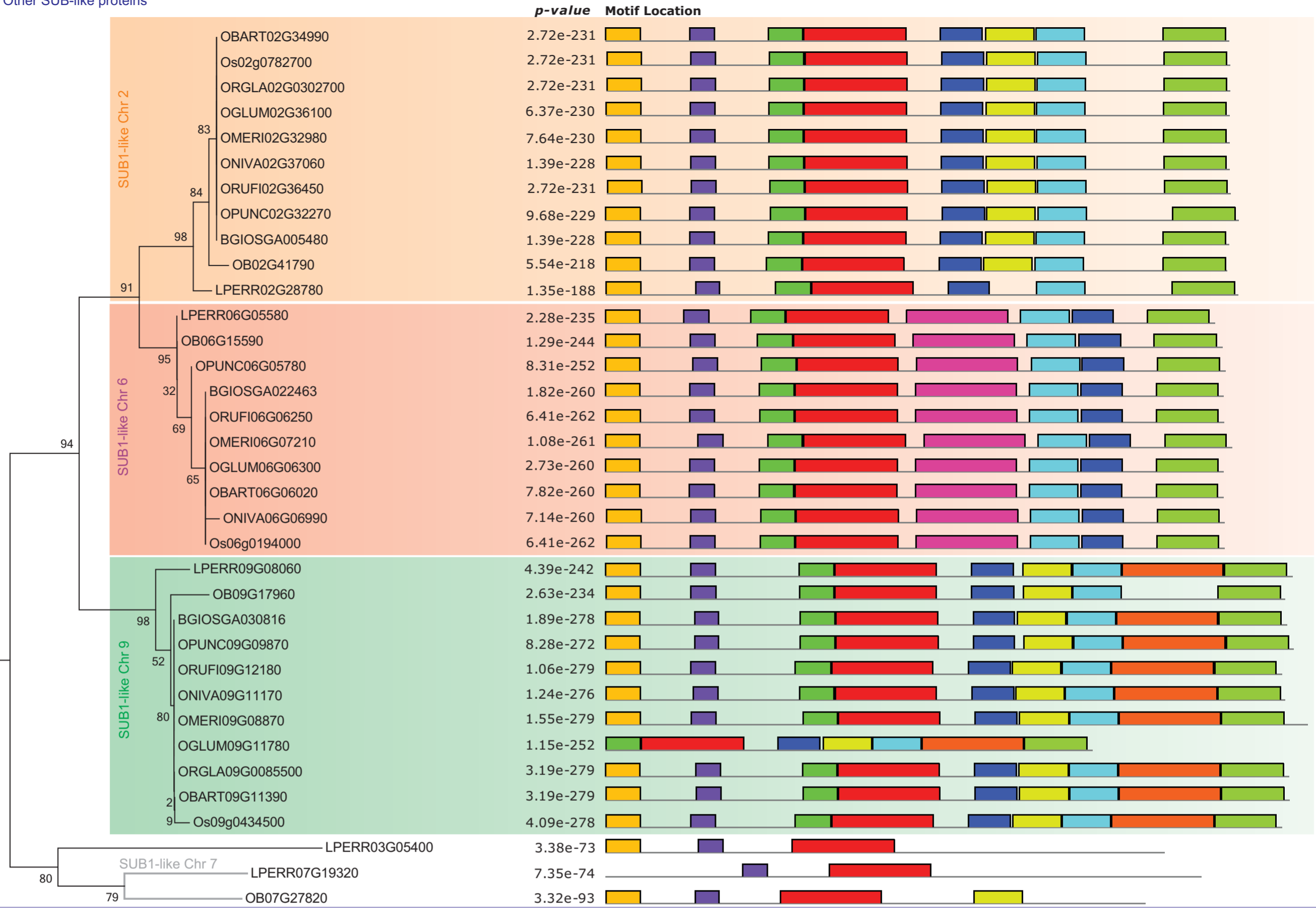

**SUB1 loci**

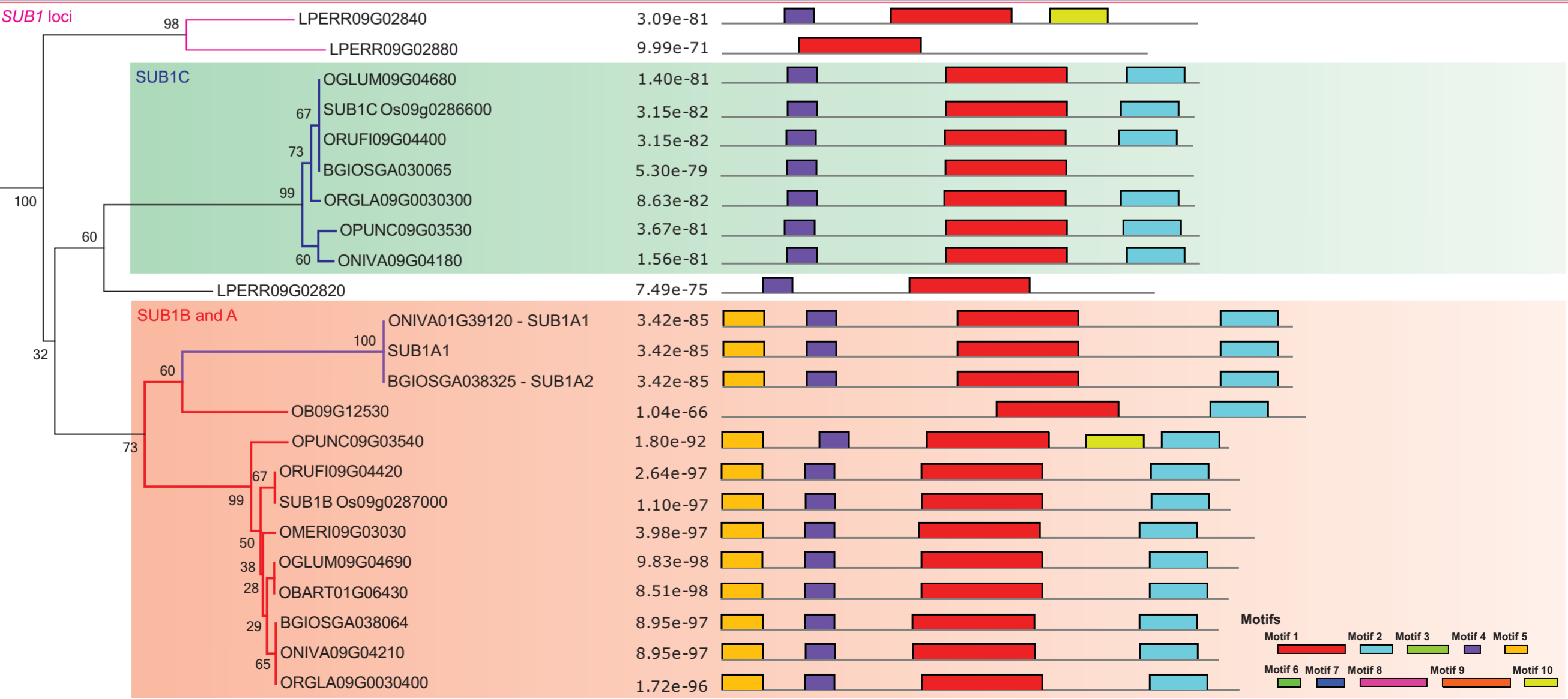

0.050

Supplement: Additional file 3: Figure S2. — Phylogenetic tree generated from the protein alignment of SUB1-like genes identified of all species analyzed, genes in red are SUB1B-like genes and green are SUB1C-like. On the right side of the tree, amino acid motifs are represented. Motif logos are available in Additional file 11: Figure S8. (PDF 1078 kb) [file 12284_2016_140_MOESM3_ESM.pdf]

Chr 1

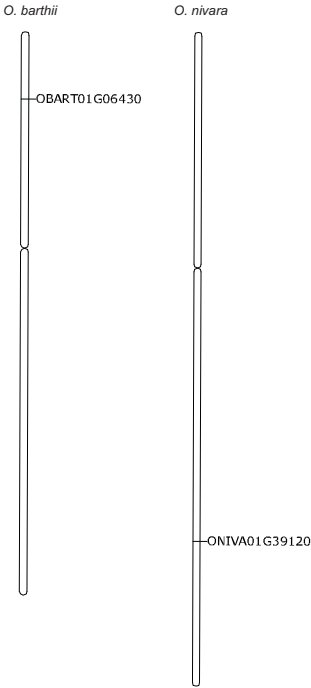

Chr 9

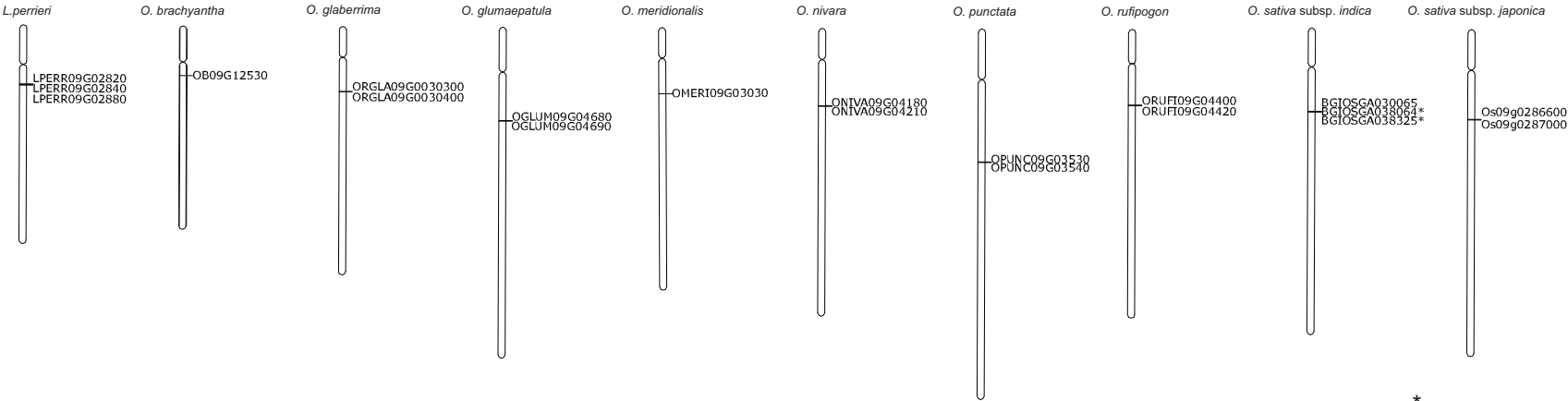

\* Scaffold not anchored to Chr 9

Supplement: Additional file 4: Figure S3. — Chromosomal location of the SUB1-like loci in each Oryza genome (chromosomes 1 and 9). (PDF 457 kb) [file 12284_2016_140_MOESM4_ESM.pdf]

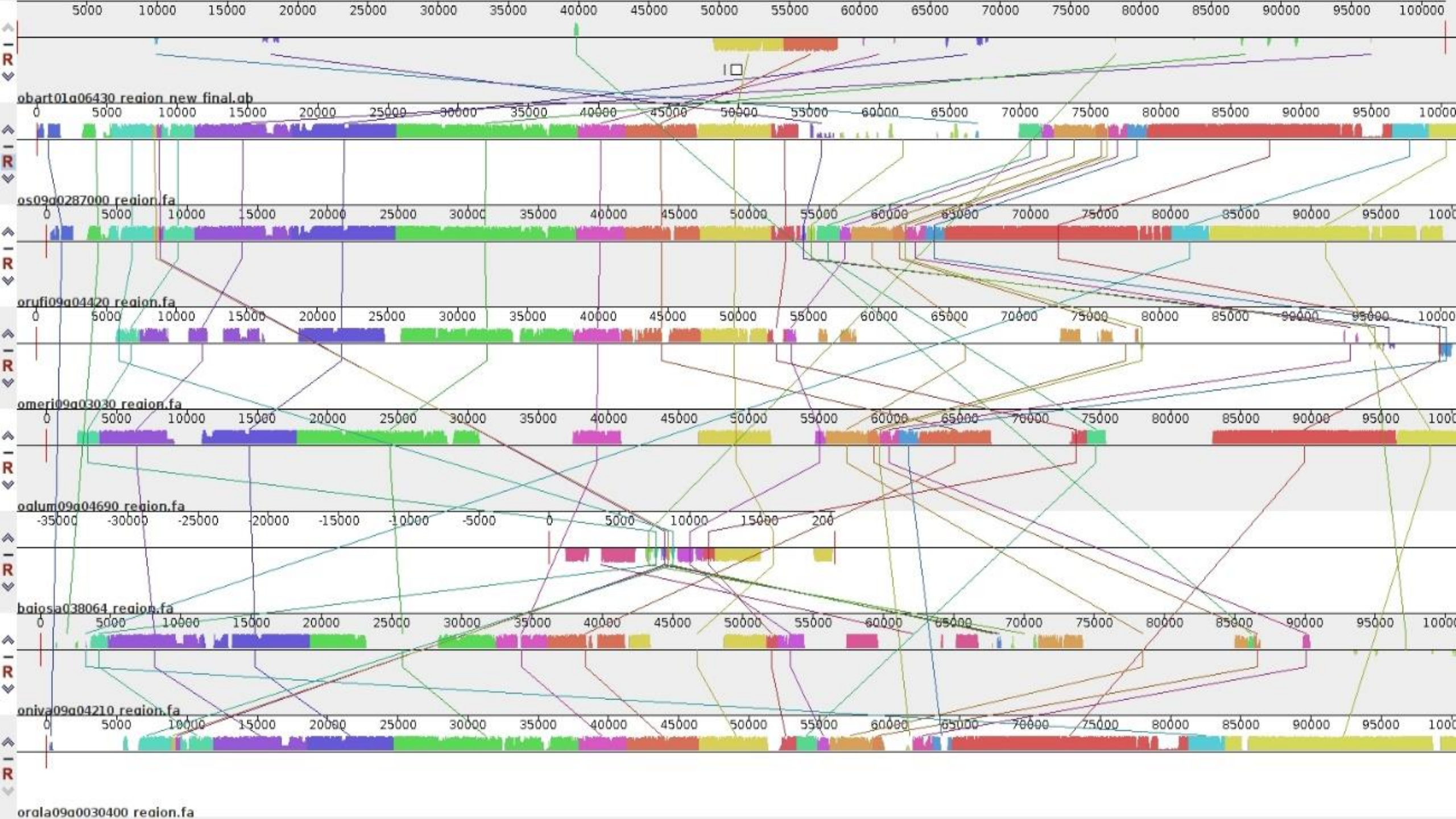

Supplement: Additional file 5: Figure S4. — Alignment of OBART01G06430 genomic region (first line) to SUB1 loci (Chr 9) regions of Oryza species of AA genome. Same colors represent collinear blocks between genomes. (PDF 174 kb) [file 12284_2016_140_MOESM5_ESM.pdf]

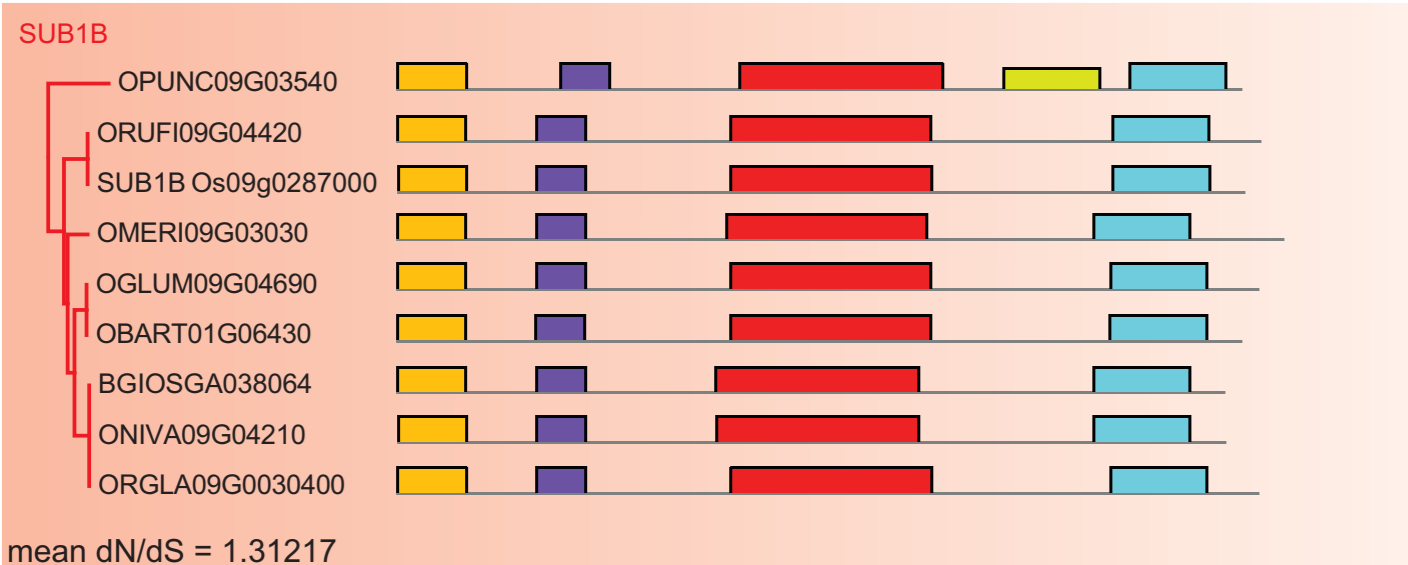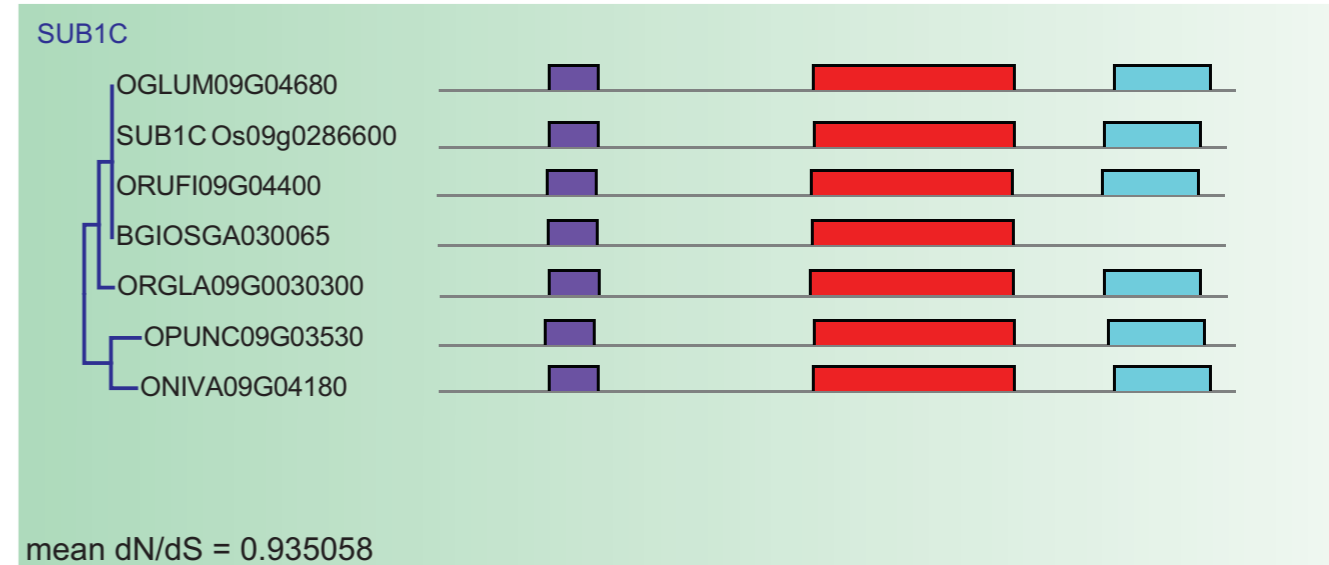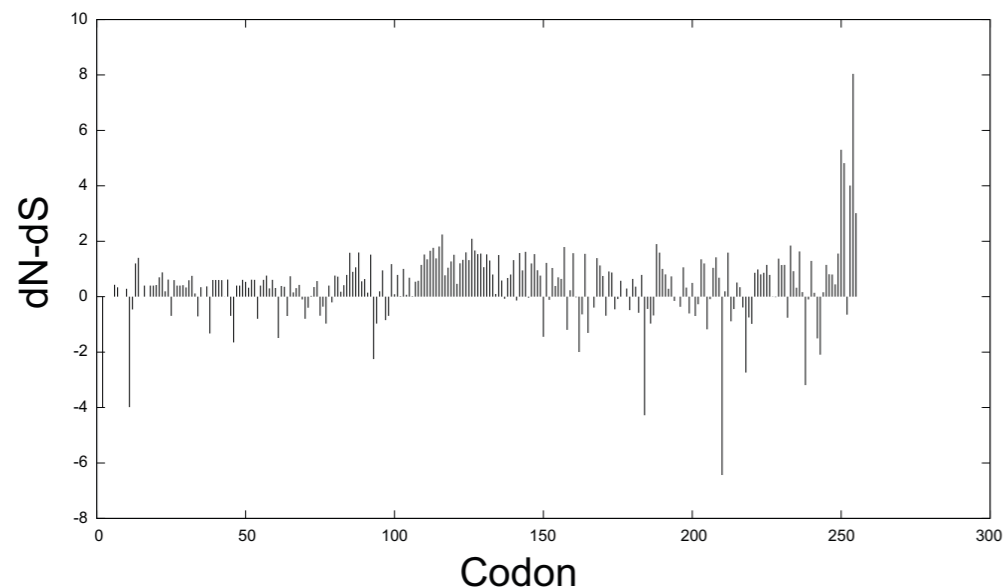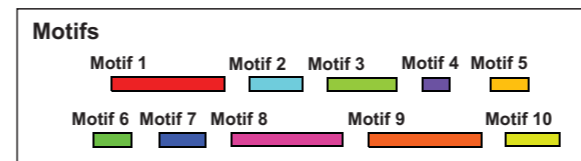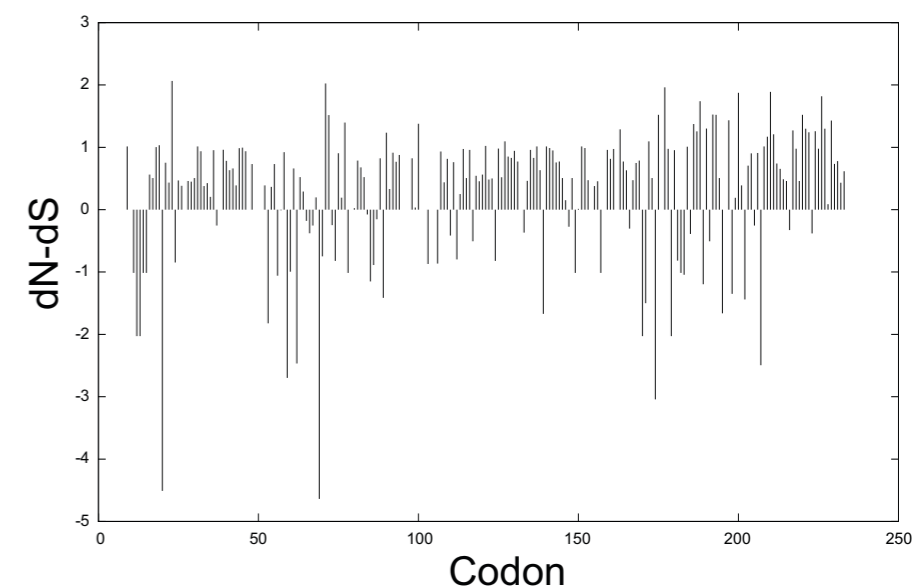

Supplement: Additional file 6: Figure S5. — Phylogenetic tree, motifs and evolutionary pressures on proteins quantified by the ratio of substitution rates at non-synonymous and synonymous sites (dN/dS) for SUB1B-like and SUB1C-like genes. (PDF 582 kb) [file 12284_2016_140_MOESM6_ESM.pdf]

SUB1B

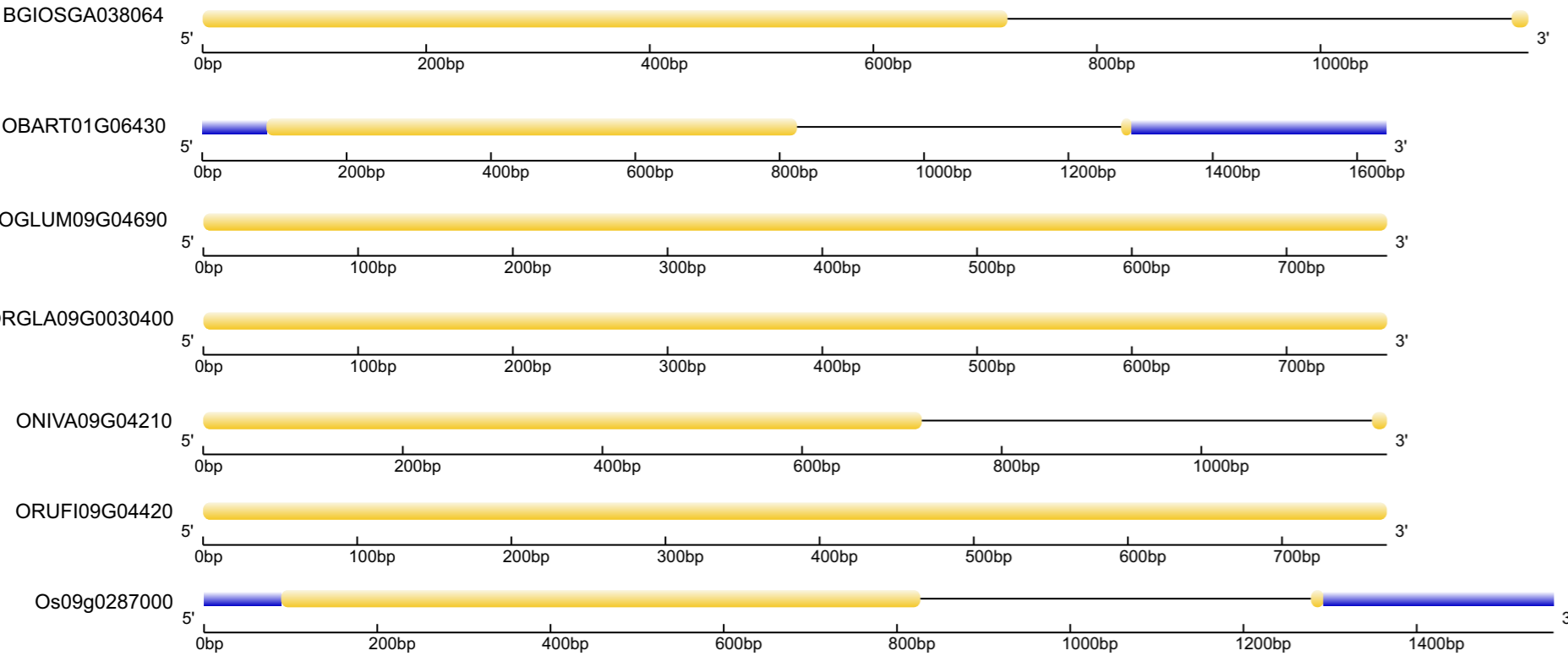

Unknown

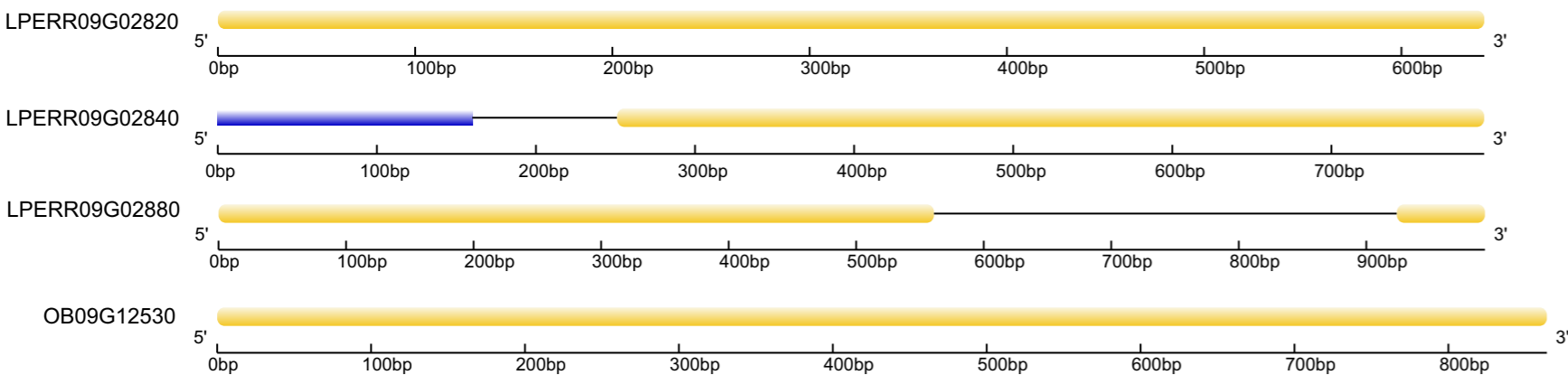

SUB1C

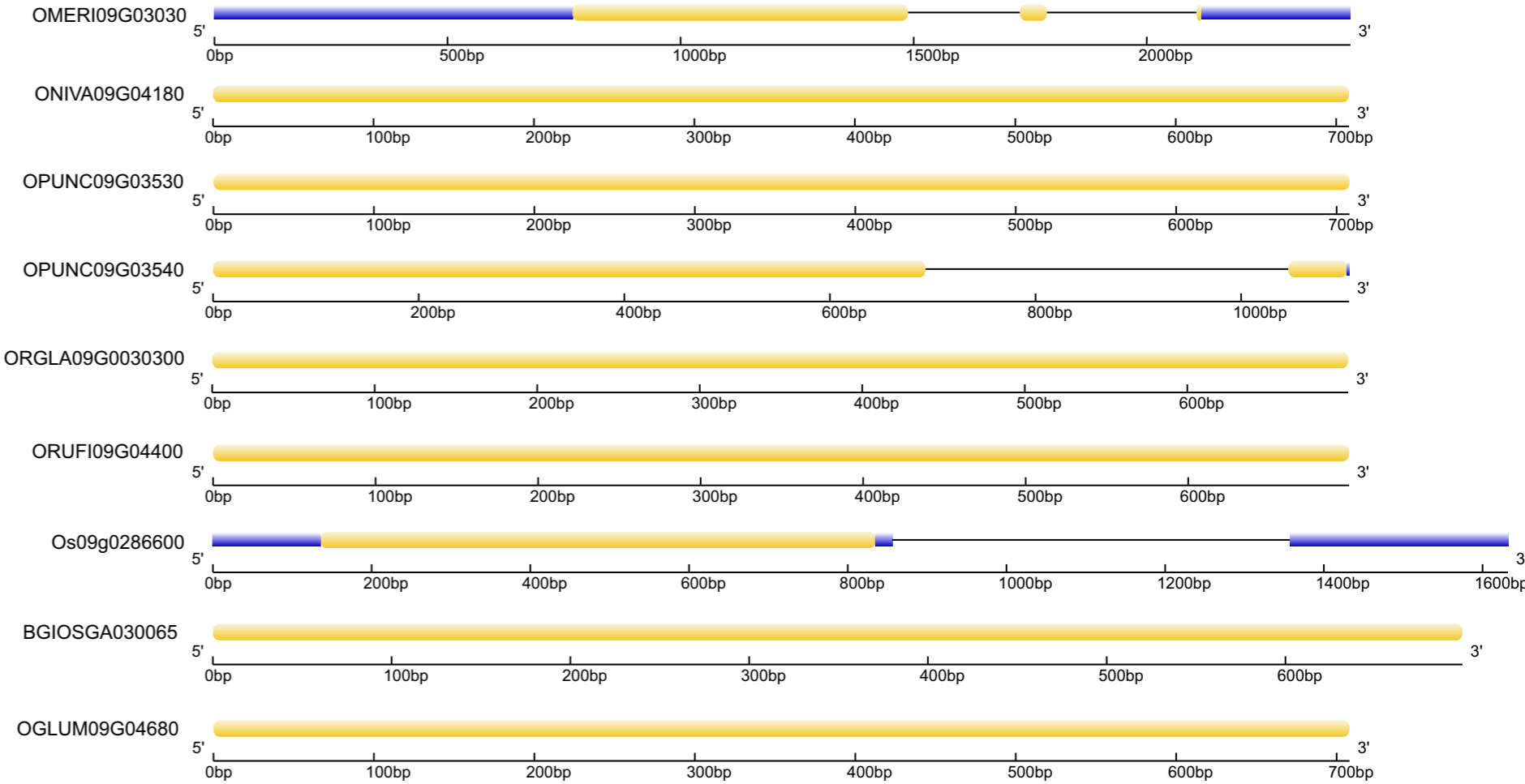

SUB1A

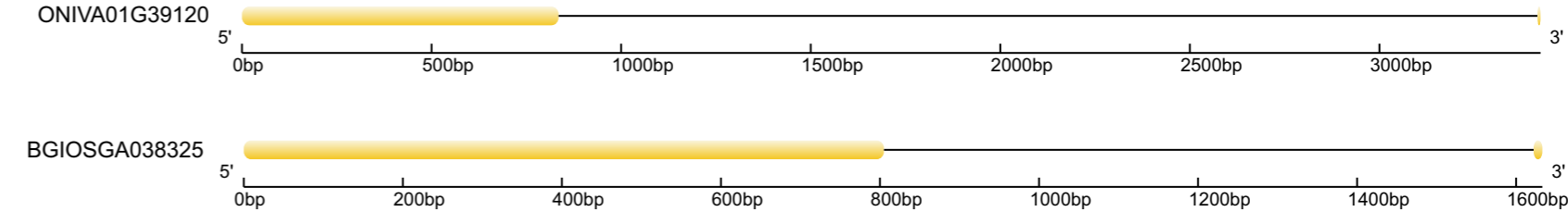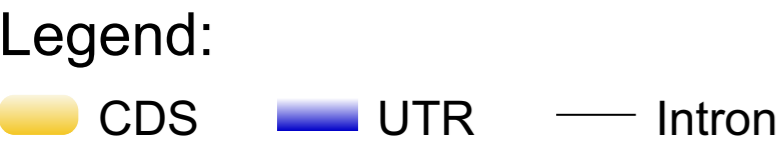

Supplement: Additional file 9: Figure S7. — Structure of the SUB1-like genes in each of the different species. (PDF 399 kb) [file 12284_2016_140_MOESM9_ESM.pdf]

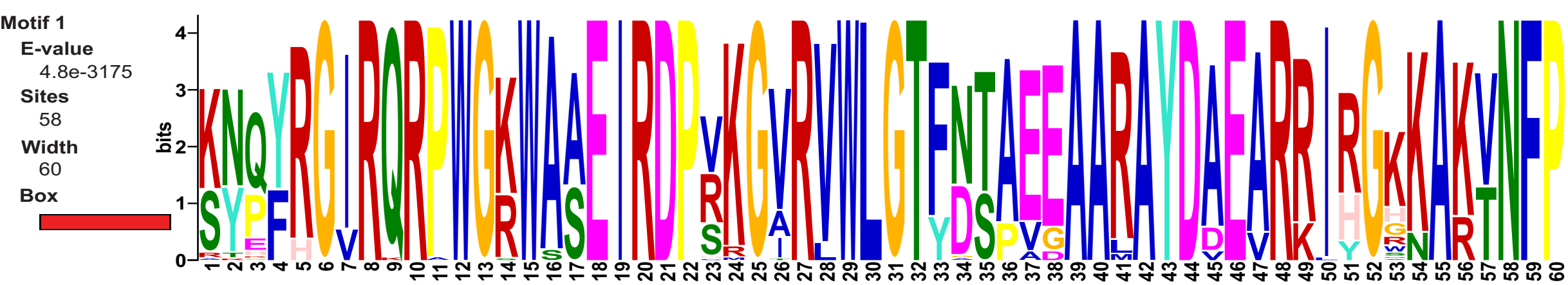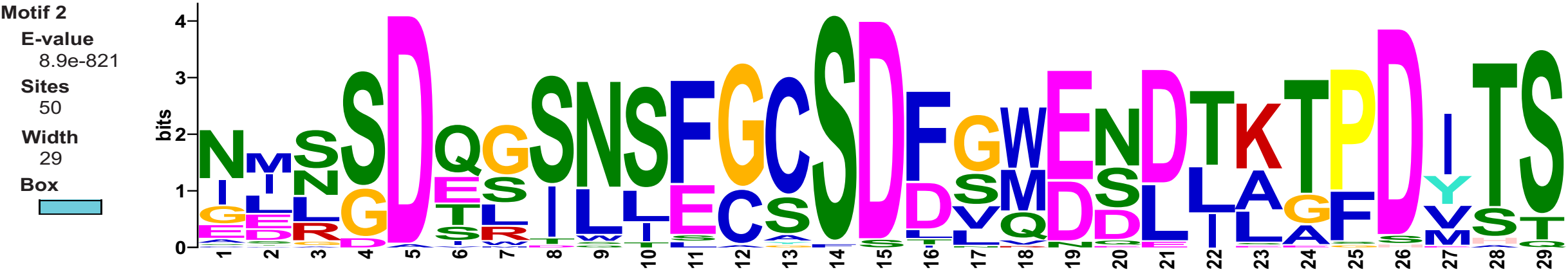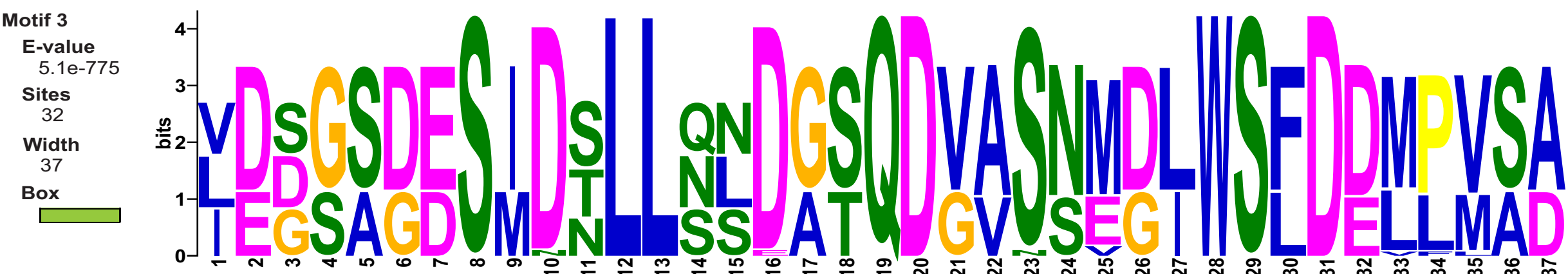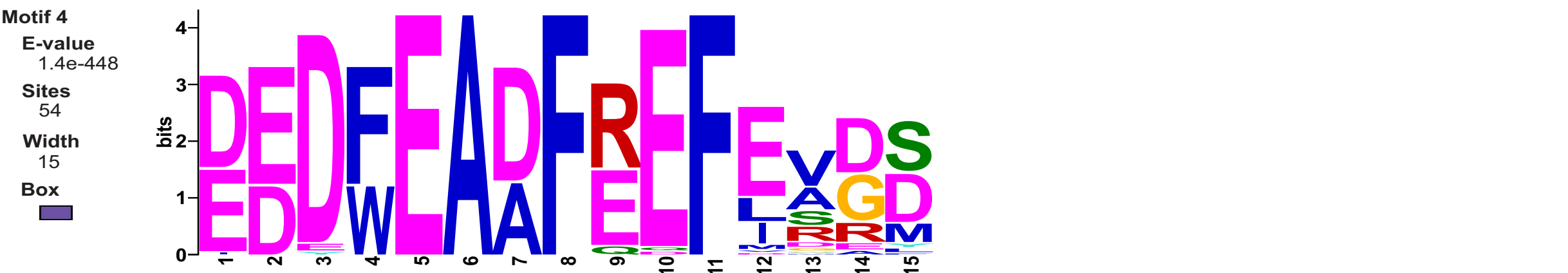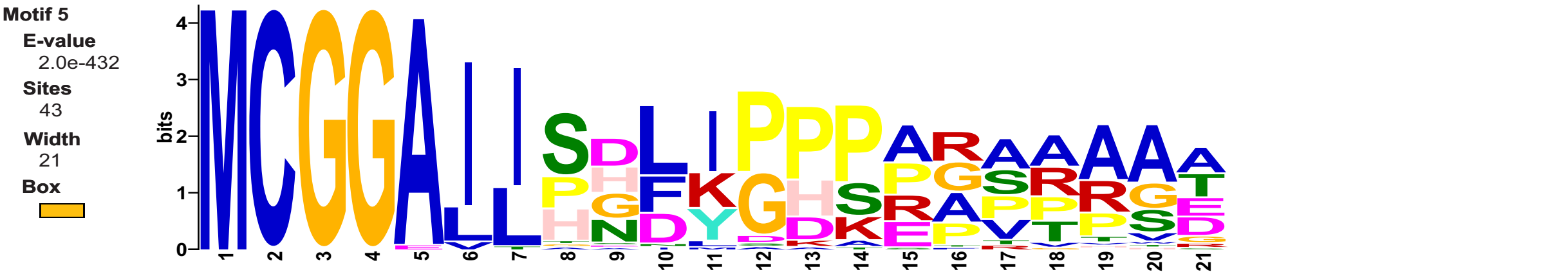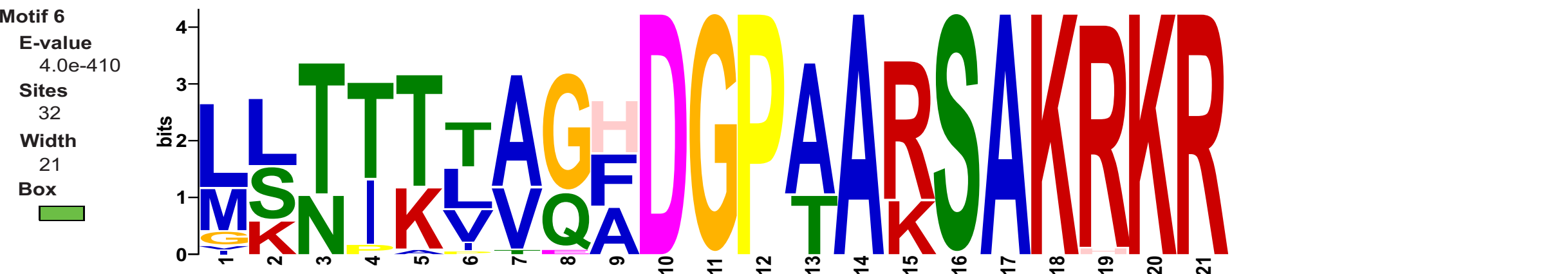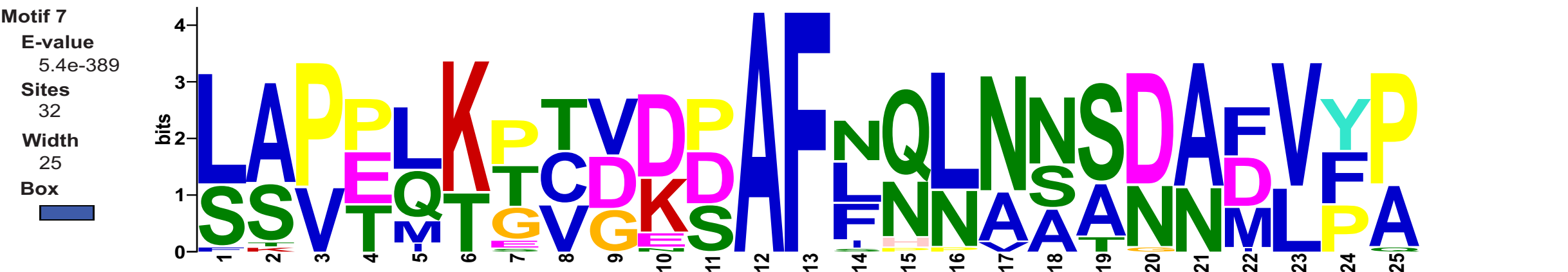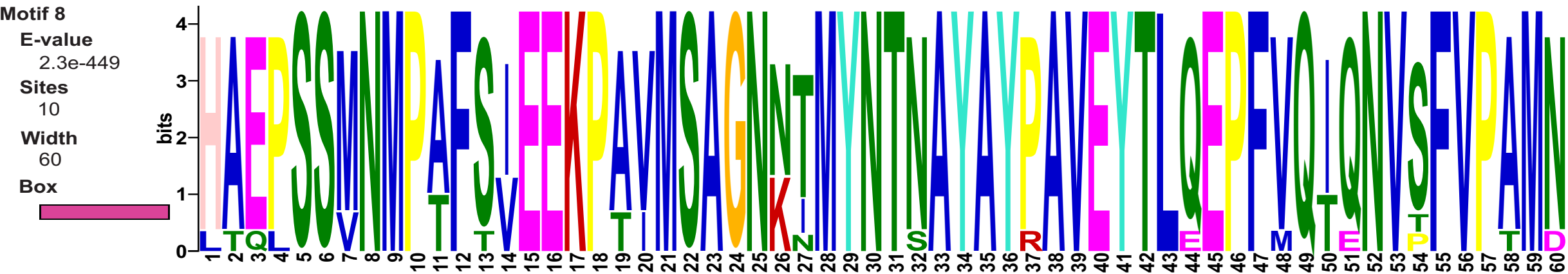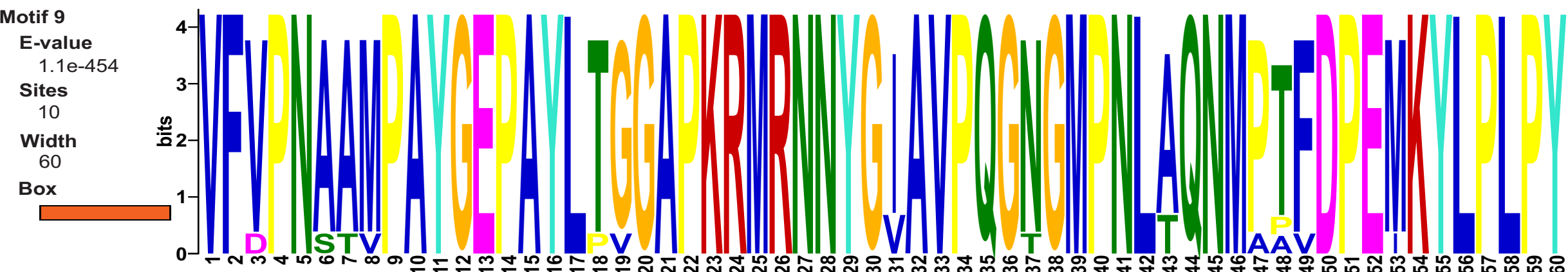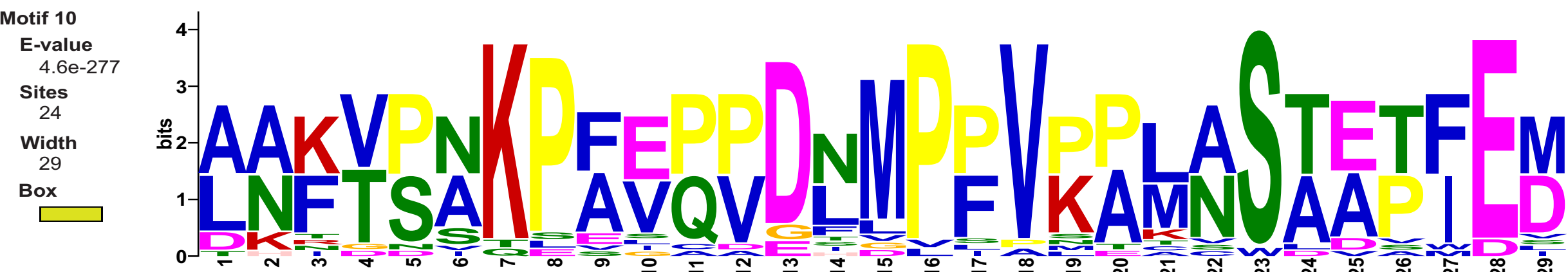

Supplement: Additional file 11: Figure S8. — Motifs found on the analysis of the amino acid sequences of SUB1-like genes. (PDF 416 kb) [file 12284_2016_140_MOESM11_ESM.pdf]
