# Supplementary material for: Evolutionary analysis of the SUB1 locus across the Oryza genomes
Source: Rice (N Y). 2017 Feb 7;10:4. doi: 10.1186/s12284-016-0140-3 (PMC5296262; doi:10.1186/s12284-016-0140-3)
Supplement: Additional file 7: Figure S6. — Alignment of the promoter region (1,500 bps upstream) of SUB1A-like genes, showing polymorphisms found when comparing SUB1A-1 and SUB1A-2 alleles. (PDF 2215 kb) [file 12284_2016_140_MOESM7_ESM.pdf]

```
                                                                 37
SUB1A-1_IR40931-26_Upstream     AGCTTATGGAGTCTACTTTCAGATGGTTTTGGTCCCCAGTCAAAATTCTTACCAAACTGC
UPSTREAM_ONIVA01G39120          AGCTTATGGAGTCTACTTTCAGATGGTTTTGGTCCCCAGTCAAAATTCTTACCAAACTGC
SUB1A-2_93-11_Upstream          AGCTTATGGAGTCTACTTTCAGATGGTTTTGGTCCCTAGTCAAAATTCTTACCAAACTGC
SUB1A-2_Teqing_Upstream         AGCTTATGGAGTCTACTTTCAGATGGTTTTGGTCCCTAGTCAAAATTCTTACCAAACTGC
                                ************************************ ***********************

                                                      85
SUB1A-1_IR40931-26_Upstream     TGGGAATCTGCAAAACAAGCCATGTATCTTATCCAGTCCGAATCTGATTTGGGTTTTGGG
UPSTREAM_ONIVA01G39120          TGGGAATCTGCAAAACAAGCCATGTATCTTATCCAGTCCGAATCTGATTTGGGTTTTGGG
SUB1A-2_93-11_Upstream          TGGGAATCTGCAAAACAAGCCACGTATCTTATCCAGTCCGAATCTGATTTGGGTTTTGGG
SUB1A-2_Teqing_Upstream         TGGGAATCTGCAAAACAAGCCACGTATCTTATCCAGTCCGAATCTGATTTGGGTTTTGGG
                                ********************** *************************************

                                                               156
SUB1A-1_IR40931-26_Upstream     CTTTGTAATTGTGTTGTGGGCTTAGCCCAAGTGGTTGTGCGCACTAGGGCAACCCTAGGA
UPSTREAM_ONIVA01G39120          CTTTGTAATTGTGTTGTGGGCTTAGCCCAAGTGGTTGTGCGCACTAGGGCAACCCTAGGA
SUB1A-2_93-11_Upstream          CTTTGTAATTGTGTTGTGGGCTTAGCCCAAGGGGTTGTGCGCACTAGGGCAACCCTAGGA
SUB1A-2_Teqing_Upstream         CTTTGTAATTGTGTTGTGGGCTTAGCCCAAGGGGTTGTGCGCACTAGGGCAACCCTAGGA
                                ******************************* ****************************

                                                                           230
SUB1A-1_IR40931-26_Upstream     CGTCCTTAATCATATTTATTCAGTAGCCGTCATCGTTTAGAGTTGGGTTTTGCTTAGAAT
UPSTREAM_ONIVA01G39120          CGTCCTTAATCATATTTATTCAGTAGCCGTCATCGTTTAGAGTTGGGTTTTGCTTAGAAT
SUB1A-2_93-11_Upstream          CGTCCTTAATCATATTTATTCAGTAGCCGTCATCGTTTAGAGTCGGGTTTTGCTTAGAAT
SUB1A-2_Teqing_Upstream         CGTCCTTAATCATATTTATTCAGTAGCCGTCATCGTTTAGAGTCGGGTTTTGCTTAGAAT
                                ******************************************* ****************

SUB1A-1_IR40931-26_Upstream     ATTCTGTCAAGAACAGTTTTGCCGCTAGATCGGTTTGTGGAACCCAAAATTAGAGTGCTT
UPSTREAM_ONIVA01G39120          ATTCTGTCAAGAACAGTTTTGCCGCTAGATCGGTTTGTGGAACCCAAAATTAGAGTGCTT
SUB1A-2_93-11_Upstream          ATTCTGTCAAGAACAGTTTTGCCGCTAGATCGGTTTGTGGAACCCAAAATTAGAGTGCTT
SUB1A-2_Teqing_Upstream         ATTCTGTCAAGAACAGTTTTGCCGCTAGATCGGTTTGTGGAACCCAAAATTAGAGTGCTT
                                ************************************************************

SUB1A-1_IR40931-26_Upstream     AATCATTCATATGCAATTGTGTTGCAATCTATCTTGTTGTTGCTTGTGTTCTTCGATTCA
UPSTREAM_ONIVA01G39120          AATCATTCATATGCAATTGTGTTGCAATCTATCTTGTTGTTGCTTGTGTTCTTCGATTCA
SUB1A-2_93-11_Upstream          AATCATTCATATGCAATTGTGTTGCAATCTATCTTGTTGTTGCTTGTGTTCTTCGATTCA
SUB1A-2_Teqing_Upstream         AATCATTCATATGCAATTGTGTTGCAATCTATCTTGTTGTTGCTTGTGTTCTTCGATTCA
                                ************************************************************

                                                        397              414
SUB1A-1_IR40931-26_Upstream     CATGCAGGGATTAGCCTTCTCGGCGAGGTCAACCGGGTTTCAGCACGGTTGATAACCGGA
UPSTREAM_ONIVA01G39120          CATGCAGGGATTAGCCTTCTCGGCGAGGTCAACCGGGTTTCAGCACGGTTGATAACCGGA
SUB1A-2_93-11_Upstream          CATGCAGGGATTAGCCTTCTCGGCAAGGTCAACCGGGTTTCGGCACGGTTGATAACCGGA
SUB1A-2_Teqing_Upstream         CATGCAGGGATTAGCCTTCTCGGCAAGGTCAACCGGGTTTCGGCACGGTTGATAACCGGA
                                ************************ **************** ******************

SUB1A-1_IR40931-26_Upstream     GGAGACGTGGTGCTACGATTGCGGGGCTCAGTTGCGTGTTCGTTCAGAAGCCGGATCGAG
UPSTREAM_ONIVA01G39120          GGAGACGTGGTGCTACGATTGCGGGGCTCAGTTGCGTGTTCGTTCAGAAGCCGGATCGAG
SUB1A-2_93-11_Upstream          GGAGACGTGGTGCTACGATTGCGGGGCTCAGTTGCGTGTTCGTTCAGAAGCCGGATCGAG
SUB1A-2_Teqing_Upstream         GGAGACGTGGTGCTACGATTGCGGGGCTCAGTTGCGTGTTCGTTCAGAAGCCGGATCGAG
                                ************************************************************

SUB1A-1_IR40931-26_Upstream     TTGTGTCGCGACTCCGCCCAAATCGACTATTTATCAATACCTATCGGAAGATCGGGACCT
UPSTREAM_ONIVA01G39120          TTGTGTCGCGACTCCGCCCAAATCGACTATTTATCAATACCTATCGGAAGATCGGGACCT
SUB1A-2_93-11_Upstream          TTGTGTCGCGACTCCGCCCAAATCGACTATTTATCAATACCTATCGGAAGATCGGGACCT
SUB1A-2_Teqing_Upstream         TTGTGTCGCGACTCCGCCCAAATCGACTATTTATCAATACCTATCGGAAGATCGGGACCT
                                ************************************************************

SUB1A-1_IR40931-26_Upstream     AACATCCCCCATCAAATAGCTATATCATTCTCAAACATTTATAGATGGATATTTAAAACC
UPSTREAM_ONIVA01G39120          AACATCCCCCATCAAATAGCTATATCATTCTCAAACATTTATAGATGGATATTTAAAACC
SUB1A-2_93-11_Upstream          AACATCCCCCATCAAATAGCTATATCATTCTCAAACATTTATAGATGGATATTTAAAACC
SUB1A-2_Teqing_Upstream         AACATCCCCCATCAAATAGCTATATCATTCTCAAACATTTATAGATGGATATTTAAAACC
                                ************************************************************

                                623
SUB1A-1_IR40931-26_Upstream     CTGCTATTTCTGCAACCGTTGCGAGCTAGCTGTCTGAACCTAAAGGCTTACGAAATGTAT
UPSTREAM_ONIVA01G39120          CTGCTATTTCTGCAACCGTTGCGAGCTAGCTGTCTGAACCTAAAGGCTTACGAAATGTAT
SUB1A-2_93-11_Upstream          CTGCTATTTCTGCAACCGTTGCGAGCTAGCTGTCTGAACCTAAAGGCTTACGAAATGTAT
SUB1A-2_Teqing_Upstream         CTACTATTTCTGCAACCGTTGCGAGCTAGCTGTCTGAACCTAAAGGCTTACGAAATGTAT
                                ** *********************************************************

                                                699
SUB1A-1_IR40931-26_Upstream     CATTTCCACGCTGGGTTATGCAGCCCTATGTGAACTAGGGAATACACCCATGTGAGAACC
UPSTREAM_ONIVA01G39120          CATTTCCACGCTGGGTTATGCAGCCCTATGTGAACTAGGGAATACACCCATGTGAGAACC
SUB1A-2_93-11_Upstream          CATTTCCACGCTGGGTCATGCAGCCCTATGTGAACTAGGGAATACACCCATGTGAGAACC
SUB1A-2_Teqing_Upstream         CATTTCCACGCTGGGTCATGCAGCCCTATGTGAACTAGGGAATACACCCATGTGAGAACC
                                **************** *******************************************

SUB1A-1_IR40931-26_Upstream     AAGCATACCACTAAGGCCATAGGTATAGATGGCCGAATCGGCTGCAAGGCCCGACCTATC
UPSTREAM_ONIVA01G39120          AAGCATACCACTAAGGCCATAGGTATAGATGGCCGAATCGGCTGCAAGGCCCGACCTATC
SUB1A-2_93-11_Upstream          AAGCATACCACTAAGGCCATAGGTATAGATGGCCGAATCGGCTGCAAGGCCCGACCTATC
SUB1A-2_Teqing_Upstream         AAGCATACCACTAAGGCCATAGGTATAGATGGCCGAATCGGCTGCAAGGCCCGACCTATC
                                ************************************************************

SUB1A-1_IR40931-26_Upstream     GGGCACGACCAAGCCCGGACCTGAGACCAGTCGGGTTACACGACCCAACGTACACGTTGG
UPSTREAM_ONIVA01G39120          GGGCACGACCAAGCCCGGACCTGAGACCAGTCGGGTTACACGACCCAACGTACACGTTGG
SUB1A-2_93-11_Upstream          GGGCACGACCAAGCCCGGACCTGAGACCAGTCGGGTTACACGACCCAACGTACACGTTGG
SUB1A-2_Teqing_Upstream         GGGCACGACCAAGCCCGGACCTGAGACCAGTCGGGTTACACGACCCAACGTACACGTTGG
                                ************************************************************

                                                                     906
SUB1A-1_IR40931-26_Upstream     GCCGGGCTGTGCCCGCCCACATGTTGCACCAACGGCCGAAGCACAATTCAATAAGACTCG
UPSTREAM_ONIVA01G39120          GCCGGGCTGTGCCCGCCCACATGTTGCACCAACGGCCGAAGCACAATTCAATAAGACTCG
SUB1A-2_93-11_Upstream          GCCGGGCTGTGCCCGCCCACATGTTGCACCAACGGCCCAAGCACAATTCAATAAGACTCG
SUB1A-2_Teqing_Upstream         GCCGGGCTGTGCCCGCCCACATGTTGCACCAACGGCCCAAGCACAATTCAATAAGACTCG
                                ************************************* **********************

SUB1A-1_IR40931-26_Upstream     GGCTGTGCCTGGCTGGCCCAAAGGCACGAAAGCCCACCGTGCTCCTCCATGGAAAAAAGT
UPSTREAM_ONIVA01G39120          GGCTGTGCCTGGCTGGCCCAAAGGCACGAAAGCCCACCGTGCTCCTCCATGGAAAAAAGT
SUB1A-2_93-11_Upstream          GGCTGTGCCTGGCTGGCCCAAAGGCACGAAAGCCCACCGTGCTCCTCCATGGAAAAAAGT
SUB1A-2_Teqing_Upstream         GGCTGTGCCTGGCTGGCCCAAAGGCACGAAAGCCCACCGTGCTCCTCCATGGAAAAAAGT
                                ************************************************************

SUB1A-1_IR40931-26_Upstream     CTATTTGCCTCTCGTATCCGGGCCATCTCTTATATGACTGAAATAAGTCTACTTTGCATT
UPSTREAM_ONIVA01G39120          CTATTTGCCTCTCGTATCCGGGCCATCTCTTATATGACTGAAATAAGTCTACTTTGCATT
SUB1A-2_93-11_Upstream          CTATTTGCCTCTCGTATCCGGGCCATCTCTTATATGACTGAAATAAGTCTACTTTGCATT
SUB1A-2_Teqing_Upstream         CTATTTGCCTCTCGTATCCGGGCCATCTCTTATATGACTGAAATAAGTCTACTTTGCATT
                                ************************************************************

SUB1A-1_IR40931-26_Upstream     CCTCATCTCTTTTGGTCGTGTCTTATACGGCTGTAATAAGTCTATTTTGGCTCCCACATC
UPSTREAM_ONIVA01G39120          CCTCATCTCTTTTGGTCGTGTCTTATACGGCTGTAATAAGTCTATTTTGGCTCCCACATC
SUB1A-2_93-11_Upstream          CCTCATCTCTTTTGGTCGTGTCTTATACGGCTGTAATAAGTCTATTTTGGCTCCCACATC
SUB1A-2_Teqing_Upstream         CCTCATCTCTTTTGGTCGTGTCTTATACGGCTGTAATAAGTCTATTTTGGCTCCCACATC
                                ************************************************************

SUB1A-1_IR40931-26_Upstream     TATTTTGCCTCCATTATTGTTGGAGAGGGCCGCCCCATCGTGCTAAGGCGGATGCCCAGG
UPSTREAM_ONIVA01G39120          TATTTTGCCTCCATTATTGTTGGAGAGGGCCGCCCCATCGTGCTAAGGCGGATGCCCAGG
SUB1A-2_93-11_Upstream          TATTTTGCCTCCATTATTGTTGGAGAGGGCCGCCCCATCGTGCTAAGGCGGATGCCCAGG
SUB1A-2_Teqing_Upstream         TATTTTGCCTCCATTATTGTTGGAGAGGGCCGCCCCATCGTGCTAAGGCGGATGCCCAGG
                                ************************************************************

SUB1A-1_IR40931-26_Upstream     CATGGCCCAAAATTCGGACCGAGGCAGCATGAGCCCGATACCAGATAGACCGAGCCATGT
UPSTREAM_ONIVA01G39120          CATGGCCCAAAATTCGGACCGAGGCAGCATGAGCCCGATACCAGATAGACCGAGCCATGT
SUB1A-2_93-11_Upstream          CATGGCCCAAAATTCGGACCGAGGCAGCATGAGCCCGATACCAGATAGACCGAGCCATGT
SUB1A-2_Teqing_Upstream         CATGGCCCAAAATTCGGACCGAGGCAGCATGAGCCCGATACCAGATAGACCGAGCCATGT
                                ************************************************************

SUB1A-1_IR40931-26_Upstream     TTGGGTTAGACCAAAACCCCGTGCCATGGGTTGGACCGTCGGGCCCCGGGCCTCTCGGCC
UPSTREAM_ONIVA01G39120          TTGGGTTAGACCAAAACCCCGTGCCATGGGTTGGACCGTCGGGCCCCGGGCCTCTCGGCC
SUB1A-2_93-11_Upstream          TTGGGTTAGACCAAAACCCCGTGCCATGGGTTGGACCGTCGGGCCCCGGGCCTCTCGGCC
SUB1A-2_Teqing_Upstream         TTGGGTTAGACCAAAACCCCGTGCCATGGGTTGGACCGTCGGGCCCCGGGCCTCTCGGCC
                                ************************************************************

SUB1A-1_IR40931-26_Upstream     AACTATAGACATAGGCACTGCAATGTTGCTTGAGAAAAAAAAAATTATAGTCACACTAGA
UPSTREAM_ONIVA01G39120          AACTATAGACATAGGCACTGCAATGTTGCTTGAGAAAAAAAAAATTATAGTCACACTAGA
SUB1A-2_93-11_Upstream          AACTATAGACATAGGCACTGCAATGTTGCTTGAGAAAAAAAAAATTATAGTCACACTAGA
SUB1A-2_Teqing_Upstream         AACTATAGACATAGGCACTGCAATGTTGCTTGAGAAAAAAAAAATTATAGTCACACTAGA
                                ************************************************************

SUB1A-1_IR40931-26_Upstream     TTGGATGTCTATTTGTTAATACTGTTAGGACAAGCACGACTTGTTCCCGCTACATCCCAC
UPSTREAM_ONIVA01G39120          TTGGATGTCTATTTGTTAATACTGTTAGGACAAGCACGACTTGTTCCCGCTACATCCCAC
SUB1A-2_93-11_Upstream          TTGGATGTCTATTTGTTAATACTGTTAGGACAAGCACGACTTGTTCCCGCTACATCCCAC
SUB1A-2_Teqing_Upstream         TTGGATGTCTATTTGTTAATACTGTTAGGACAAGCACGACTTGTTCCCGCTACATCCCAC
                                ************************************************************

SUB1A-1_IR40931-26_Upstream     TCCGATATACAAAGTGTATTTCGACCGCCCCTTGGCCTTCCAGCCTATATATATCAACAA
UPSTREAM_ONIVA01G39120          TCCGATATACAAAGTGTATTTCGACCGCCCCTTGGCCTTCCAGCCTATATATATCAACAA
SUB1A-2_93-11_Upstream          TCCGATATACAAAGTGTATTTCGACCGCCCCTTGGCCTTCCAGCCTATATATATCAACAA
SUB1A-2_Teqing_Upstream         TCCGATATACAAAGTGTATTTCGACCGCCCCTTGGCCTTCCAGCCTATATATATCAACAA

SUB1A-1_IR40931-26_Upstream     CTTCTACCC
UPSTREAM_ONIVA01G39120          CTTCTACCC
SUB1A-2_93-11_Upstream          CTTCTACCC
SUB1A-2_Teqing_Upstream         CTTCTACCC
                                *********
```
