# Supplementary material for: Evolutionary analysis of the SUB1 locus across the Oryza genomes
Source: Rice (N Y). 2017 Feb 7;10:4. doi: 10.1186/s12284-016-0140-3 (PMC5296262; doi:10.1186/s12284-016-0140-3)
Supplement: Additional file 14: — Methods. (DOCX 24 kb) [file 12284_2016_140_MOESM14_ESM.docx]

**METHODS**

**DNA/RNA sequencing and gene prediction**

DNA sequences used in this study were obtained from the complete genome sequencing of *O.* *rufipogon* (Cultivar: W1943; Gramene accession: PRJEB4137), *O.* *nivara* (IRGC:100897; AWHD00000000), *O.* *glumaepatula* (GEN1233; ALNU00000000), *O.* *glaberrima* (IRGC:96717; ADWL00000000), *O.* *barthii* (IRGC:105608; ABRL00000000), *O.* *meridionalis* (OR44 (W2112); ALNW00000000), *O.* *punctata* (IRGC:105690; AVCL00000000), *O.* *brachyantha* (IRGC:101232; AGAT00000000) and *Leersia* *perrieri* (A. Camus) Launert (IRGC:105164; ALNV00000000). Sequencing, assembly and annotation of these species are part of the IOMAP initiative in which Next Generation Sequencing (NGS) was used for obtaining both genomic and transcriptomic sequences. Raw RNAseq data used to enhance our gene annotation should be made available soon, along with the study of Stein et al (unpublished).

**Prediction of centromere position**

The centromere position of chrs.1 and 9 of each species has been predicted through a BLASTn (<https://blast.ncbi.nlm.nih.gov/Blast.cgi?PAGE_TYPE=BlastSearch>) using the centromere region of *O.* *sativa* ssp *japonica*: chr. 1 (16610866..17243770) and chr. 9 (2749793..3043847) as queries. The subject aligned region of the best BLAST hit was used as centromere for graphic construction.

**Identification of SUB1 genes in the *Oryza* genus**

Proteins of *SUB1* genes obtained from Fukao *et al*. (2009) were used as queries for BLASTp analysis (Altschul et al., 1990) ([https://blast.ncbi.nlm.nih.gov/](https://blast.ncbi.nlm.nih.gov/Blast.cgi?PAGE_TYPE=BlastSearch)) against the predicted gene sequences of the nine species under analysis (Table S4). Hits were selected based on e-value (< 1e^-24^). After BLAST, existence of *Apetala2* (AP2) domain was verified using Pfam (Finn et al., 2014) (<http://pfam.xfam.org/>) and then sequences were aligned using ClustalOmega (Thompson et al., 1994) (<http://www.ebi.ac.uk/Tools/msa/clustalo/>) to confirm the presence of *SUB1* signature amino acids.

**Phylogenetic analysis**

Sequences of *O.* *sativa* *SUB1* genes were obtained from the Rice Annotation Project Database (RAP-DB; see URLs) (*SUB1B* and *SUB1C* of *O.* *sativa* ssp *japonica*) and from the National Center for Biotechnology Information (NCBI; <http://www.ncbi.nlm.nih.gov/>) (*SUB1A*, *SUB1B* and *SUB1C* of *O.* *sativa* ssp *indica*). Phylogenetic analyses were performed using the software Molecular Evolutionary Genetics Analysis 7 – MEGA 7 (Tamura et al., 2013). The sequences were aligned by ClustalW using Gonnet weight matrix, and the tree was constructed using the neighbor-joining method (Saitou and Nei, 1987) with 10,000 bootstrap replicates. Motif identification was conducted using the Multiple Motif In Elicitation version 4.11.1 (MEME; <http://meme-suite.org/tools/meme>) (Bailey et al., 2009) considering a maximum number of motifs ranging from 10. The ratio of synonymous/non-synonymous mutations (Ka/Ks or dN/dS) was calculated by Datamonkey Webserver (Delport et al., 2010). The gene structure (data available in Table S5) was visualized by GSDS 2.0 (Hu et al., 2015).

**Translocation Event Analysis**

To understand the origin of *ONIVA01G39120* and *OBART01G06430*, which could have occurred due to translocation events from *SUB1* genes originally in Chr 9, an alignment of regions corresponding to 50 Kb upstream and 50 Kb downstream of these genes to 50 Kb upstream and 50 Kb downstream of the *SUB1* loci (Chr 9) was performed in every *Oryza* species, using both NCBI blast and Mauve (Altschul et al., 1990; Darling et al., 2010). RiTE database (Copetti et al., 2015) was used to verify if this possible event occurred due to the translocation of transposable elements (TEs).

**Co-occurrence of Transcription Factor Binding Sites**

The search of transcription factor binding sites (TFBS) inside the promoter region (-1,500 bps) of the analyzed genes was determined using the “Promoter Analysis” tool of PlantPAN 2.0 (Chang et al., 2008) (<http://plantpan2.itps.ncku.edu.tw/>). Here rice and arabidopsis databases of TFBSs were used with standard settings.

**REFERENCES**

Altschul SF, Gish W, Miller W, Meyers EW, Lipman DJ (1990) Basic Local Alignment Search Tool. Journal of Molecular Biology 215:403-410.

Bailey TL, Boden M, Buske FA, Frith M, Grant CE, Clementi L, Ren J, Li WW, Noble WS (2009) MEME SUITE: tools for motif discovery and searching. Nucleic Acids Research 37:W202-W208. doi: 10.1093/nar/gkp335

Chang W-C, Lee T-Y, Huang, H-D, Huang, H-Y, Pan R-L (2008) PlantPAN: Plant promoter analysis navigator, for identifying combinatorial cis-regulatory elements with distance constraint in plant gene groups. BMC Genomics 9. doi: 10.1186/1471-2164-9-561

Copetti D, Zhang J, El Baidouri M, Gao D, Wang J, Barghini E, Cossu RM, Angelova A, Maldonado L CE, Roffler S, Ohyanagi H, Wicker T, Fan C, Zuccolo A, Chen M, Costa de Oliveira A, Han B, Henry R, Hsing YI, Kurata N, Wang W, Jackson SA, Panaud O, Wing RA (2015) RiTE database: a resource database for genus-wide rice genomics and evolutionary biology. BMC genomics 16:538. doi: 10.1186/s12864-015-1762-3

Darling AE, Mau B, Perna NT (2010) progressiveMauve: multiple genome alignment with gene gain, loss and rearrangement. PloS one 5:e11147. doi: 10.1371/journal.pone.0011147

Delport W, Poon AF, Frost SD, Kosakovsky Pond SL (2010) Datamonkey 2010: a suite of phylogenetic analysis tools for evolutionary biology. Bioinformatics 26:2455-2457. doi: 10.1093/bioinformatics/btq429

Finn RD, Bateman A, Clements J, Coggill P, Eberhardt RY, Eddy SR, Heger A, Hetherington K, Holm L, Mistry J, et al. (2014). Pfam: the protein families database. Nucleic acids research 42:D222-230. doi: 10.1093/nar/gkt1223

Hu B, Jin J, Guo AY, Zhang H, Luo J, Gao G (2015) GSDS 2.0: an upgraded gene feature visualization server. Bioinformatics 31:1296-1297. doi: 10.1093/bioinformatics/btu817

Saitou N, Nei M. (1987) The neighbor-joining method: a new method for reconstructing phylogenetic trees. Mol Biol Evol 4:406-425.

Stein JC et al (The International Oryza Map Alignment Consortium, unpublished) Genomes of 11 rice relatives unveil genetic conservation, turnover and innovation across the genus *Oryza*. submitted (Nature).

Tamura K, Stecher G, Peterson D, Filipski A., Kumar S (2013). MEGA6: Molecular Evolutionary Genetics Analysis version 6.0. Mol Biol Evol 30:2725-2729. doi: 10.1093/molbev/mst197

Thompson JD, Higgins DG, Gibson TJ (1994) CLUSTAL W: improving the sensitivity of progressive multiple sequence alignment through sequence weighting, position-specific gap penalties and weight matrix choice. Nucleic acids research 22:4673-4680.
