# Supplementary material for: Evolutionary analysis of the SUB1 locus across the Oryza genomes
Source: Rice (N Y). 2017 Feb 7;10:4. doi: 10.1186/s12284-016-0140-3 (PMC5296262; doi:10.1186/s12284-016-0140-3)
Supplement: Additional file 2: Figure S1. — Alignment of the AP2 domains of SUB1-like genes in rice relatives. Signature amino acids specific to B2 subgroup of ERF proteins are highlighted. (PDF 403 kb) [file 12284_2016_140_MOESM2_ESM.pdf]

```
                               13   18
LPERR03G05400         FRGIRQRPWGKWAAEIRDPHKGTRVWLGTFNTPEEAARAYDVEARRLRGSKAKVNF
LPERR07G19320         YRGIRQRPWGKWAAEIRDPSKGVRVWLGTFATAEEAAMAYDVEARRIRGKKAKVNF
OB07G27820            YRGIRQRPWGKWAAEIRDPSKGVRVWLGTFGTAEEAAMAYDVEARRIRGKKAKVNF
LPERR09G08060         YRGIRQRPWGKWAAEIRDPSKGVRVWLGTYNTAEEAARAYDAEARKIRGKKAKVNF
BGIOSGA030816         YRGIRQRPWGKWAAEIRDPSKGVRVWLGTYNTAEEAARAYDAEARKIRGKKAKVNF
OPUNC09G09870         YRGIRQRPWGKWAAEIRDPSKGVRVWLGTYNTAEEAARAYDAEARKIRGKKAKVNF
ORUFI09G12180         YRGIRQRPWGKWAAEIRDPSKGVRVWLGTYNTAEEAARAYDAEARKIRGKKAKVNF
ONIVA09G11170         YRGIRQRPWGKWAAEIRDPSKGVRVWLGTYNTAEEAARAYDAEARKIRGKKAKVNF
OMERI09G08870         YRGIRQRPWGKWAAEIRDPSKGVRVWLGTYNTAEEAARAYDAEARKIRGKKAKVNF
OGLUM09G11780         YRGIRQRPWGKWAAEIRDPSKGVRVWLGTYNTAEEAARAYDAEARKIRGKKAKVNF
ORGLA09G0085500       YRGIRQRPWGKWAAEIRDPSKGVRVWLGTYNTAEEAARAYDAEARKIRGKKAKVNF
OB09G17960            YRGIRQRPWGKWAAEIRDPSKGVRVWLGTYNTAEEAARAYDAEARKIRGKKAKVNF
OBART09G11390         YRGIRQRPWGKWAAEIRDPSKGVRVWLGTYNTAEEAARAYDAEARKIRGKKAKVNF
Os09g0434500          YRGIRQRPWGKWAAEIRDPSKGVRVWLGTYNTAEEAARAYDAEARKIRGKKAKVNF
ONIVA06G06990         FRGIRQRPWGKWAAEIRDPCKGVRVWLGTFNSPEEAARAYDAEARRIRGKKAKVNF
LPERR02G28780         FRGIRQRPWGKWAAEIRDPRKGVRVWLGTYNSAEEAARAYDAEARRIRGKKAKVNF
LPERR06G05580         FRGIRQRPWGKWAAEIRDPRKGVRVWLGTFNSPEEAARAYDAEARRIRGKKAKVNF
BGIOSGA022463         FRGIRQRPWGKWAAEIRDPRKGVRVWLGTFNSPEEAARAYDAEARRIRGKKAKVNF
OPUNC06G05780         FRGIRQRPWGKWAAEIRDPRKGVRVWLGTFNSPEEAARAYDAEARRIRGKKAKVNF
ORUFI06G06250         FRGIRQRPWGKWAAEIRDPRKGVRVWLGTFNSPEEAARAYDAEARRIRGKKAKVNF
OMERI06G07210         FRGIRQRPWGKWAAEIRDPRKGVRVWLGTFNSPEEAARAYDAEARRIRGKKAKVNF
OGLUM06G06300         FRGIRQRPWGKWAAEIRDPRKGVRVWLGTFNSPEEAARAYDAEARRIRGKKAKVNF
OB06G15590            FRGIRQRPWGKWAAEIRDPRKGVRVWLGTFNSPEEAARAYDAEARRIRGKKAKVNF
OBART06G06020         FRGIRQRPWGKWAAEIRDPRKGVRVWLGTFNSPEEAARAYDAEARRIRGKKAKVNF
Os06g0194000          FRGIRQRPWGKWAAEIRDPRKGVRVWLGTFNSPEEAARAYDAEARRIRGKKAKVNF
BGIOSGA005480         FRGIRQRPWGKWAAEIRDPRKGVRVWLGTFNSAEEAARAYDAEARRIRGKKAKVNF
OPUNC02G32270         FRGIRQRPWGKWAAEIRDPRKGVRVWLGTFNSAEEAARAYDAEARRIRGKKAKVNF
ORUFI02G36450         FRGIRQRPWGKWAAEIRDPRKGVRVWLGTFNSAEEAARAYDAEARRIRGKKAKVNF
ONIVA02G37060         FRGIRQRPWGKWAAEIRDPRKGVRVWLGTFNSAEEAARAYDAEARRIRGKKAKVNF
OMERI02G32980         FRGIRQRPWGKWAAEIRDPRKGVRVWLGTFNSAEEAARAYDAEARRIRGKKAKVNF
OGLUM02G36100         FRGIRQRPWGKWAAEIRDPRKGVRVWLGTFNSAEEAARAYDAEARRIRGKKAKVNF
ORGLA02G0302700       FRGIRQRPWGKWAAEIRDPRKGVRVWLGTFNSAEEAARAYDAEARRIRGKKAKVNF
OB02G41790            FRGIRQRPWGKWAAEIRDPRKGVRVWLGTFNSAEEAARAYDAEARRIRGKKAKVNF
OBART02G34990         FRGIRQRPWGKWAAEIRDPRKGVRVWLGTFNSAEEAARAYDAEARRIRGKKAKVNF
Os02g0782700          FRGIRQRPWGKWAAEIRDPRKGVRVWLGTFNSAEEAARAYDAEARRIRGKKAKVNF
OPUNC09G03540         YRGVRQRPWGRWASEIRDPVKGARVWLGTYDTAVEAARAYDAEARRIHGAKARTNF
OMERI09G03030         YRGVRQRPWGRWASEIRDPVKGARVWLGTFDTAVEAARAYDAEARRIHGQKARTNF
BGIOSGA038064         YRGVRQRPWGRWASEIRDPVKGARVWLGTFDTAAEAARAYDAEARRIHGHKARTNF
ONIVA09G04210         YRGVRQRPWGRWASEIRDPVKGARVWLGTFDTAAEAARAYDAEARRIHGHKARTNF
ORGLA09G0030400       YRGVRQRPWGRWASEIRDPVKGARVWLGTFDTAAEAARAYDAEARRIHGHKARTNF
ORUFI09G04420         YRGVRQRPWGRWASEIRDPVKGARVWLGTFDTAVEAARAYDAEARRIHGHKARTNF
OGLUM09G04690         YRGVRQRPWGRWASEIRDPVKGARVWLGTFDTAVEAARAYDAEARRIHGHKARTNF
OBART01G06430         YRGVRQRPWGRWASEIRDPVKGARVWLGTFDTAVEAARAYDAEARRIHGHKARTNF
SUB1B_Os09g0287000    YRGVRQRPWGRWASEIRDPVKGARVWLGTFDTAVEAARAYDAEARRIHGHKARTNF
BGIOSGA030065         YRGIRQRPWGRWASEIRDPVKGIRVWLGTFDTAEGAARAYDDEVRRIYGGNAKTNF
ORUFI09G04400         YRGIRQRPWGRWASEIRDPVKGIRVWLGTFDTAEGAARAYDDEVRRIYGGNAKTNF
ONIVA09G04180         YRGIRQRPWGRWASEIRDPVKGIRVWLGTFDTAEGAARAYDDEVRRIYGGNAKTNF
OGLUM09G04680         YRGIRQRPWGRWASEIRDPVKGIRVWLGTFDTAEGAARAYDDEVRRIYGGNAKTNF
ORGLA09G0030300       YRGIRQRPWGRWASEIRDPVKGIRVWLGTFDTAEGAARAYDDEVRRIYGGNAKTNF
SUB1C_Os09g0286600    YRGIRQRPWGRWASEIRDPVKGIRVWLGTFDTAEGAARAYDDEVRRIYGGNAKTNF
OPUNC09G03530         YRGIRQRPWGRWASEIRDPVKGIRVWLGTFDTAEGAARAYDDEVRRIYGSNAKTNF
LPERR09G02820         YRGIRQRPWGRWASEIRDPVRGVRVWLGTFDTAEDAARAYDAEVRRIHGRKAKTNF
LPERR09G02840         YHGIRKRPWGKWASEIRDPVMGVRLWLGTFDTAEDAARAYDAEARRIHGRNAKTNF
LPERR09G02880         YHGIRQRAWGKWASEIRDPVRGVRLWLGTFDTAEDAARAYDAEARRIHGRKAKTNF
SUB1A
BGIOSGA038325         YHGIRQRPWGRWSSEIRDPVKGVRLWLGTFDTAVEAALAYDAEARRIHGWKARTNF
ONIVA01G39120         YHGIRQRPWGRWSSEIRDPVKGVRLWLGTFDTAVEAALAYDAEARRIHGWKARTNF
SUB1A1                YHGIRQRPWGRWSSEIRDPVKGVRLWLGTFDTAVEAALAYDAEARRIHGWKARTNF
OB09G12530            YHGIRQRPWGSWASEIRDPVRGVRVWLGTFDTAEEAALAYDAEARRIHGRKARTNF
                      ::*:*:* ** *::*****  * ****:   ** *** *.*:: * :*:.**
```
